# The spatiotemporal profile of *Dendrobium huoshanense* and functional identification of *bHLH* genes under exogenous MeJA using comparative transcriptomics and genomics

**DOI:** 10.3389/fpls.2023.1169386

**Published:** 2023-05-10

**Authors:** Xiaomei He, Wenwu Zhang, Irfan Ali Sabir, Chunyan Jiao, Guohui Li, Yan Wang, Fucheng Zhu, Jun Dai, Longyun Liu, Cunwu Chen, Yingyu Zhang, Cheng Song

**Affiliations:** ^1^ Anhui Engineering Laboratory for Conservation and Sustainable Utilization of Traditional Chinese Medicine Resources, Anhui Engineering Research Center for Eco-agriculture of Traditional Chinese Medicine, College of Biological and Pharmaceutical Engineering, West Anhui University, Lu’an, China; ^2^ School of Life Science, Anhui Agricultural University, Hefei, China; ^3^ Department of Plant Science, School of Agriculture and Biology, Shanghai Jiao Tong University, Shanghai, China; ^4^ College of Life Sciences, Hefei Normal University, Hefei, China; ^5^ School of Bioengineering, Hefei Technology College, Hefei, China; ^6^ Henan Key Laboratory of Rare Diseases, Endocrinology and Metabolism Center, The First Affiliated Hospital, and College of Clinical Medicine of Henan University of Science and Technology, Luoyang, China

**Keywords:** *Dendrobium huoshanense*, bHLH transcription factor, JA signaling, abiotic stress, genomics

## Abstract

**Introduction:**

Alkaloids are one of the main medicinal components of *Dendrobium* species. *Dendrobium* alkaloids are mainly composed of terpene alkaloids. Jasmonic acid (JA) induce the biosynthesis of such alkaloids, mainly by enhancing the expression of JA-responsive genes to increase plant resistance and increase the content of alkaloids. Many JA-responsive genes are the target genes of bHLH transcription factors (TFs), especially the MYC2 transcription factor.

**Methods:**

In this study, the differentially expressed genes involved in the JA signaling pathway were screened out from *Dendrobium huoshanense* using comparative transcriptomics approaches, revealing the critical roles of basic helix-loop-helix (bHLH) family, particularly the MYC2 subfamily.

**Results and discussion:**

Microsynteny-based comparative genomics demonstrated that whole genome duplication (WGD) and segmental duplication events drove *bHLH* genes expansion and functional divergence. Tandem duplication accelerated the generation of *bHLH* paralogs. Multiple sequence alignments showed that all bHLH proteins included bHLH-zip and ACT-like conserved domains. The MYC2 subfamily had a typical bHLH-MYC_N domain. The phylogenetic tree revealed the classification and putative roles of bHLHs. The analysis of *cis*-acting elements revealed that promoter of the majority of *bHLH* genes contain multiple regulatory elements relevant to light response, hormone responses, and abiotic stresses, and the *bHLH* genes could be activated by binding these elements. The expression profiling and *q*RT-PCR results indicated that *bHLH* subgroups IIIe and IIId may have an antagonistic role in JA-mediated expression of stress-related genes. *DhbHLH20* and *DhbHLH21* were considered to be the positive regulators in the early response of JA signaling, while *DhbHLH24* and *DhbHLH25* might be the negative regulators. Our findings may provide a practical reference for the functional study of *DhbHLH* genes and the regulation of secondary metabolites.

## Introduction

bHLH TFs play pivotal roles in the JA signaling pathways, which are crucial for the coordination of the plant’s adaptive response to abiotic stresses (Goossens et al., 2017; Altmann et al., 2020). *D. huoshanense*, a highly prized traditional Chinese medicine, has always been subjected to a variety of obstacles over the course of artificial domestication. In response, the JA-mediated *bHLH* genes are systematically tuned to regulate the resistance to abiotic stress and the biosynthesis of defensive substance (Major et al., 2017; Song et al., 2022a). bHLH TFs are involved in a variety of physiological processes, such as sexual development, nutrition, and basal metabolism (Feller et al., 2011; Zhang et al., 2020; Liu et al., 2021a). A total of 162, 167, 95, and 152 *bHLH* genes have been reported in *Arabidopsis thaliana*, *Oryza sativa*, *Vitis vinifera*, and *Solanum lycopersicum*, respectively. The conserved domains of bHLH TFs commonly contain two functionally distinct motifs, one basic and one helix-loop-helix (HLH) region (Liu et al., 2021b). The HLH domain at the C-terminus is approximately 40 amino acids long and aids in the formation of some homodimeric and heterodimeric protein complexes (Howe et al., 2018). Earlier evolutionary analyses demonstrated that plant bHLH proteins could be subdivided into 26 subfamilies, 20 of which were present in the ancestor of vascular and bryophyte plants (Fan et al., 2021; Hao et al., 2021). In *A. thaliana, AtbHLH42* generally controls the biosynthesis of anthocyanins (Baudry et al., 2004; Song et al., 2021a; Zhou et al., 2020). *AtbHLH122* overexpression was found to have higher salt tolerance and anaerobic stress than its wild relatives (Liu et al., 2014). The expression level of *AtbHLH92* is significantly enhanced in response to NaCl, drought, and cold stresses (Zander et al., 2020; Jiang et al., 2009). *AtbHLH38* and *AtbHLH39* influenced the iron ion metabolism (Fan et al., 2021). *AtbHLH112* is a transcriptional regulator in the stress signaling pathway, despite the inhibitory effect on plant development. (Hao et al., 2021). The homologs *bHLH068* from *O. sativa* and *bHLH112* from *A. thaliana* have antagonistic effects on the mediation of flowering initiation (Chen et al., 2017).

MYC TFs are one of the subfamilies of the bHLH family, including bHLH subgroups IIId, IIIe, and IIIf (Cao et al., 2022). In *A. thaliana*, *bHLH* subgroup IIIe members include *AtMYC2*, *AtMYC3*, *AtMYC4*, and *AtMYC5*. AtMYC2-mediated expression of JA-responsive genes is finely tuned by the JAZ proteins and the coactivator mediator complex subunit MED25 (An et al., 2017; Liu et al., 2019). MYC2 could interact with the majority of JAZ proteins, depending on the JID domain (Fernández-Calvo et al., 2011; Du et al., 2017). The bHLH subclade IIIe was associated positively with the systemic JA response (Qi et al., 2015a). In contrast, bHLH subclade IIId has a negative function in numerous JA-mediated responses (Song et al., 2013). Several studies have demonstrated that the bHLH subclade IIIe is essential for JA signaling. MYC2 orchestrated a precise module of COI1/JAZ/MYC2 to participate in the JA signaling (Peñuelas et al., 2019). MYC2, MYC3, and MYC4 homologs were reported to form some homodimers or heterodimers and bind to the G-box of targeted genes, despite having some redundancy with MYC2 (Hao et al., 2021). MYC2, MYC3, and MYC4 activated JA-induced leaf senescence by binding the promoter of *SAG29* gene (Zheng et al., 2017). However, the bHLH subgroup IIIb binded to its promoter to inhibit the expression and slowed down the senescence process (Qi et al., 2015b). MYC5 modulated JA-mediated herbivore defenses and affected JA-mediated pathogen resistance in plants (Song et al., 2017).

*D. huoshanense* is a semi-shaded perennial orchid plant, as well as a rare and endangered traditional Chinese medicine. When *Dendrobium* plant is exposed to diverse harsh conditions, high temperature, drought, and UV-B have impacts on its yield and accumulation of medicinal substances (Song et al., 2020; Pan et al., 2023). In normal growth conditions, JA is at a low level; when subjected to external stress, the JA content of the seedlings rapidly increased, which activates JA-responsive genes to increase resistance to environmental stimuli (Zhu et al., 2017). JA also induced the expression of key enzymes in the terpenoid biosynthesis pathway and raised terpene alkaloid content in a short period of time (Song et al., 2022b). The *bHLH* genes have been widely investigated in both model plants and medicinal plants. Until now, little is known about the molecular control mechanism of the bHLH TFs in *D. huoshanense*, particularly the MYC2 in response to JA signal transduction. The response of *D. huoshanense* to stress involves intricate physiological and molecular regulatory processes. Discovering the regulatory factors associated with stress resistance is critical for understanding Dendrobium’s resistance mechanism and guiding the genetic improvement of stress resistance (Wu et al., 2019). In this study, JA-induced comparative transcriptome sequencing was used to identify the crucial genes that respond to JA signaling in different spatio-temporal trajectories. It revealed the core and unique regulatory role of *bHLH* family genes, particularly the MYC2 and JAM subfamilies, in the JA regulatory network. A total of 83 *bHLHs* were identified from the *D. huoshanense* genome. Successive analyses, including the gene mapping, the gene structure, the gene motifs, the phylogenetic tree construction, the conserved domain analysis, the *cis*-acting element analysis, the microcollinearity analysis, *etc.*, were further performed. Our study may provide a scientific reference for the functional research of *bHLH* under abiotic stress in *Dendrobium* species.

## Materials and methods

### Plant material and growing conditions

The cultivated seedlings were collected from the Anhui Plant Cell Engineering Center of West Anhui University. The coordinates for sampling were 31° 45′ 58′′ N and 116° 29′ 09′′ E. *D. huoshanense* has a growth temperature of 25 ± 2°C, a humidity of 60–70%, a light culture of 16 hours, and a dark culture of 8 hours. Plants with stable growth rates and growth years were chosen. The leaves were sprayed with water and a 50 mmol/L MeJA solution, respectively. Time intervals are 0.25 hours, 0.5 hours, 1 hour, 2 hours, 4 hours, 8 hours, and 16 hours. The control and MeJA-treated leaves at different time points were collected and added into liquid nitrogen for quick freezing, and then put into a -80°C refrigerator for later use.

### cDNA library construction and data quality control

Total RNA was extracted with Trizol method, and the steps were referred to in the previous experimental protocol (Song et al., 2021b). Firstly, the total RNA is treated with mRNA enrichment, and those with the polyA tail is enriched with OligodT magnetic beads. The DNA probe is digested with DNaseI, and the desired RNA is obtained after purification. PCR is performed with specific primers from a cDNA library that has been amplified. The raw image data obtained by sequencing is converted into raw reads through base calling, and low-quality, adapter contamination, and reads with a high content of unknown base N are removed. The filtered data is called “clean reads.” Filter with the filtering software SOAPnuke (v1.4.0). After obtaining the clean reads, HISAT (v2.1.0) was applied to align the clean reads to the reference genome sequence (Kim et al., 2015). Bowtie2 (v2.2.5) was used to map the clean reads to the reference genome, and then gene and transcript expression value was calculated based on RSEM (Li and Dewey, 2011; Langmead and Salzberg, 2012). The overall analysis of transcriptome sequencing was performed, including coverage and distribution of transcripts (Bjornson et al., 2021). The overall analysis of this transcriptome sequencing was carried out, including statistics such as sequencing saturation. All the original transcript data that was removed from redundancy was uploaded to the GSA database of the National Genomics Data Center (https://ngdc.cncb.ac.cn/gsa/), and the released accession was CRA006607.

### Gene structure and variation analysis

Transcript reconstruction was performed on each sample using StringTie (v1.0.4) (Pertea et al., 2015). The Cuffmerge was used to integrate the reconstruction information of all samples. In order to identify novel transcripts, the integrated transcripts were compared to reference annotation data using Cuffcompare (Trapnell et al., 2012). The CPC (v0.9-r2) was used to predict the protein-coding new transcripts, and finally the new transcripts were combined to the reference gene sequence to obtain complete sequence (Kang et al., 2017). The rMATS (v3.2.5) was employed to detect differentially spliced genes among different samples and splicing events of different groups (Shen et al., 2014).Benjamini algorithm was applied to correct the *p* value to calculate the *FDR* value (Benjamini and Hochberg, 1995).

### Gene annotation and quantitative analysis

Firstly, the GO, NR, and KEGG orthology databases were used for functional annotation of all genes. The getorf software was used to detect the ORF of unigene (http://emboss.sourceforge.net/apps/cvs/emboss/apps/getorf.html). Then, hmmsearch command was used to align the ORF to the transcription factor conserved domain to characterize the transcription factor family according to the PlantTFDB database (http://plntfdb.bio.uni-potsdam.de/v3.0/). The DIAMOND (v0.8.31) software was used to retrieve the plant resistance gene database PRGdb (http://prgdb.crg.eu/) (Sanseverino et al., 2009; Buchfink et al., 2014). The running parameters for DIAMOND: –evalue 1e-5 –outfmt 6 –max -target-seqs 1 –more-sensitive. Screening parameters: query coverage >= 50%, identity >= 40%. Gene expression levels of the individual samples were calculated using RSEM (v1.2.8) software (Li and Dewey, 2011). Using the cor function, the Pearson correlation between each two samples was calculated to get the correlation matrix. PCA analysis was performed using the princomp function, and the graphics were drawn using the ggplot2 package. The expression level distribution map was made based on the FPKM value of each sample. The number of co-expressed and differentially expressed genes among different samples was obtained through the Venn diagram of the expression level between groups. Time series analysis was used to figure out how genes were expressed at different points in time, and Mfuzz (v2.34.0) was used to cluster genes with similar expression patterns together (Kumar & Futschik, 2007).

### Analysis of differentially expressed genes

The DEG detection between each of the two groups was undertaken using the DEseq2 method (Love et al., 2014). Hierarchical clustering analysis was performed using the pheatmap function. According to the GO annotation results and the classic classification, the differential genes by function were classified using the phyper function for enrichment analysis. Based on the KEGG annotation results and the classic classification, the DEGs were converged into biological pathways. The phyper function in the R software was used to perform enrichment analysis, calculate the p-value, and then perform *FDR* correction on the *p*-value. Parameters with *Q*-values greater than 0.05 were considered significantly enriched.

### Identification of the *bHLH* gene in *D. huoshanense*

The Hidden Markov Model of the HLH domain (PF00010) obtained from the Pfam database was used to identify bHLHs in *D. huoshanense* genome under the accession PRJNA597621 (Han et al., 2020). The Pfam database was then used to see if all candidates had the HLH domain. Compared with the databases with an e-value of 0.001, the HMM profile was used as a query to identify all HLH domains in *D. huoshanense*. Pfam (http://pfam.xfam.org/search/), SMART (http://smart.embl-heidelberg.de/), and InterPro (http://www.ebi.ac.uk/interpro/search/sequence/) were used to confirm all candidate DhbHLHs. The EnsemblPlants (http://plants.ensembl.org/index.html) database was used to obtain the genome sequence files of *A. thaliana* and *O. sativa*. The genome sequence and coding sequence file of *D. nobile* and *D. chrysotoxum* were obtained from the NCBI database (https://www.ncbi.nlm.nih.gov/genome). The physical characteristics of bHLHs, including the protein sequence length, gene ID, and chromosome location, were obtained from the *D. huoshanense* genome. The ExPASy Bioinformatics Resource Portal (https://web.expasy.org) was used to predict the molecular weight and isoelectric point of each WRKY protein (Gasteiger et al., 2003). All putative redundant sequences were discarded based on the sequence alignments.

### Identification of the conserved domain and synteny analysis of *bHLH* genes

The bHLH proteins of *A. thaliana, O. sativa, D. nobile*, and *D. chrysotoxum* were compared using multiple alignment. The WebLogo (http://weblogo.berkeley.edu) service was used to investigate the conserved domains. The GeneDoc (https://www.psc.edu/biomed/genedoc) software was used to color the conserved amino acids based on protein homology. The MCScanX (https://github.com/wyp1125/MCScanX) software was used to ascertain the microsynteny between each two species, including *A. thaliana, O. sativa, D. nobile*, and *D. chrysotoxum*, based on the genome files (Wang et al., 2012).

### The phylogenetic analysis of *bHLH* genes

At first, multiple sequence alignments of the bHLH proteins were performed using the ClustalX 2.11 with the default parameters (Gao et al., 2017; Liu et al., 2017; Wang et al., 2017). Then, the phylogenetic tree of bHLHs in *A. thaliana* and *D. huoshanense* was constructed using maximum-likelihood methods. The candidate protein sequence was aligned using MEGA(v 6.0). (Tamura et al., 2013). The phylogenetic tree was then created using the IQ-TREE program. The best-fit model was optimized as JTT+F+R7 to produce a more trustworthy WRKY classification. The running parameter was “iqtree.exe -s./bidui.fas -m JTT+F+R7 -bb 1000-alrt 1000-nt AUTO”.

### The chromosome location, conserved motif and gene structure analysis of *DhbHLHs*

The chromosomal location of *DhbHLH* genes was visualized using the TBtools software (Chen et al., 2020). The gene density was retrieved from the gff file. The chromosome mapping was built using TBtools. The motif pattern and gene structure were also visualized using TBtools. The conserved motifs were identified by searching the candidate proteins with the MEME suite tool (https://meme-suite.org/). The number of motifs was limited to 20, while the width of the motifs ranged from 6 to 200. In terms of gene structure analysis, the gff files were used to visualize the UTR, exon, and intron regions.

### The *cis*-acting element analysis of *DhbHLHs*

To discover the *cis*-acting elements in the promoter, the 2000 bp upstream sequence of the promoter was extracted from the *D. huoshanense* genome (Carvalho et al., 2015; Song et al., 2021b). Initially, the promoters of all genes were extracted using the GFF/GTF sequence extractor tool from TBtools software. The *cis*-acting elements were then extracted using the rapid fasta extractor and filter tools. The PlantCARE service (http://bioinformatics.psb.ugent.be/webtools/plantcare/html/) was performed to obtain these responsive elements.

### Expression profile analysis of *DhbHLHs* under different MeJA treatments

The differential expressed profiling analysis was performed under the control group and seven MeJA treatment groups to determine which *bHLH* family members are strongly up- and down regulated by JA signaling. The fragments per kilobase of exon model per million mapped fragments (FPKM) value was used to calculate the TPM value. Following that, the Salmon tool was used to analysis expression levels based on our RNA-seq datasets (Patro et al., 2017). The expression level for replicates in the same group is the average of all samples. The FPKM of all unigenes was utilized to create an expression profile of differentially expressed genes (Wang et al., 2019). The normalized FPKM (log_2_ (FPKM)) of all unigenes was used for the hierarchical clustering and expression analyses of *DhbHLH* genes (Song et al., 2022c).

### The *q*RT-PCR analysis of the main *DhbHLHs* genes

Total RNA was extracted from *Dendrobium* leaves of the blank and MeJA treatment groups using the RNAprep Pure Plant Kit (Takara, Japan) (CK and T1–T7). The RNA content and purity were determined using an ultra-micro spectrophotometer. The quantitative analysis was performed using the 7500 series real-time fluorescence quantitative PCR (Bio-RAD, America). Twelve candidates were obtained from the genome. The PrimeScript RT reagent kit was used to transform the total RNA from the above samples into cDNA (Takara, Japan). For *q*RT-PCR test, a 20 μl reaction system was used: 10 μl SYBR Premix Ex Taq II, 2 μl cDNA, 0.8 μl *DhbHLH*-RT-F and *DhbHLH*-RT-R, 0.4 μl ROX Reference Dye II, and 6 μl dH_2_O. The PCR reaction program refers to the previous method (Song et al., 2021a). The 2^-ΔΔCt^ method was used to calculate the relative expression with *actin* gene as the reference (Livak and Schmittgen, 2001).

## Results

### The reference genome comparison, novel gene identification, and gene structure analysis

**Figure 1:**
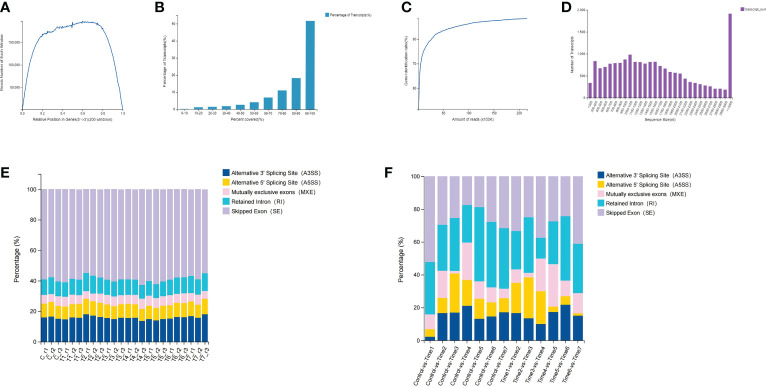
Data quality assessment and gene structure analysis. **(A)** Distribution of clean reads in transcripts; **(B)** Read coverage of all transcripts; **(C)** Sequencing reads saturation curve; **(D)** Length of all transcripts; **(E)** Alternative shearing of all transcripts **(F)** Differential alternative splicing analysis of transcripts.

In this study, the BGISEQ-500 platform was used for next-generation transcriptome sequencing, and a total of 8 groups of 24 samples were used for the high-throughput sequencing. On the basis of the published *D. huoshanense* genome, the average alignment rate of the samples with the genome was 92.29%, while the average alignment rate of the gene set was 71.66% (**Tables S1**, **S2**). Through a comprehensive quality evaluation of the sequencing data and transcript coverage, the results showed that the reads were evenly distributed in various regions of the transcript, the relative position of genes ranged from 0.2 to 0.8 (**Figure 1A**). According to the genome comparison results, the transcript coverage per sample were counted. The results showed that more than 50% of transcripts had read coverage above 90%, while 20% of transcripts had read coverage between 80% and 90% (**Figure 1B**). By calculating the saturation curve of the detected genes, the sample had enough sequencing data (**Figure 1C**). The length of most transcripts ranges from 500 to 2000 nucleotides, but when the sequence length exceeds 3000 nucleotides, the number of transcripts increased dramatically (**Figure 1D**). A total of 8,969 new genes were predicted; a total of 27,264 genes were identified, of which 18,418 were known genes and 8,846 new genes; a total of 41,069 new transcripts were identified, of which 17,374 belonged to novel alternative splicing isoforms of protein-coding genes, with 8,969 transcripts belonging to novel protein-coding genes and the remaining 14,726 belonging to long non-coding RNA (**Table S3**). Through gene structure and variant analysis, the type and number of alternative splicing of all genes were determined (**Figure 1F**). The results showed that nearly 60% of the alternative splicing events belonged to skipped exon (SE), followed by alternative 3’ splicing site (A3SS), and mutually exclusive exons (MXE) events accounted for less than 10% (**Figure 1E**; **Table S4**). Differential analysis of alternative splicing of different samples showed that Control-vs-Time1, Time6-vs-Time7 and Time3-vs-Time4 had more SEs. Except for the Time3-vs-Time4 group, the proportion of retained intron (RI) events was the same in each group. Interestingly, the Control-vs-Time1 group contained fewer A3SS and alternative 5’ splicing site (A5SS) events.

### Gene annotation based on public database alignment

**Figure 2:**
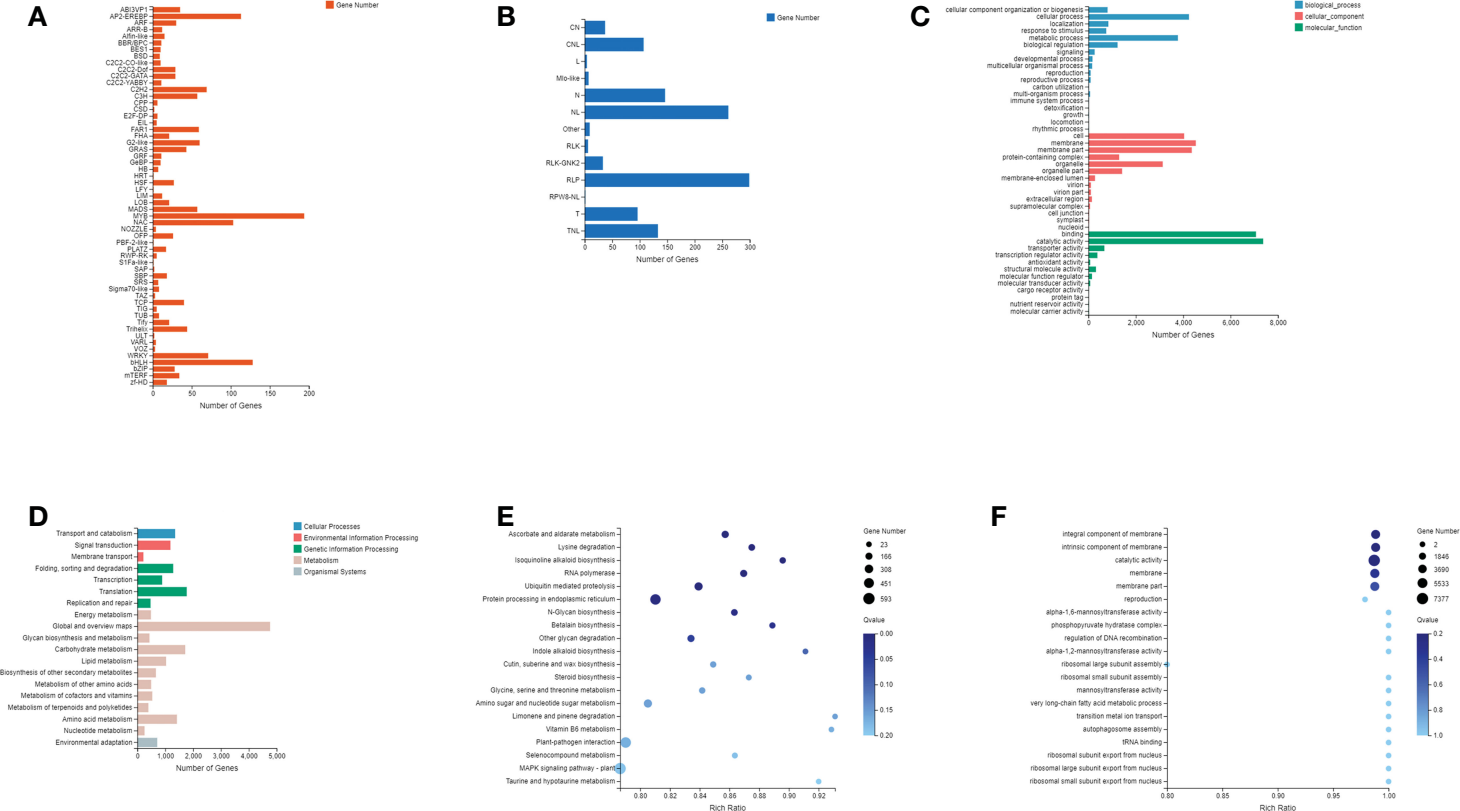
Annotation of identified genes and novel genes. **(A)** Family and number of all transcription factors identified; **(B)** Domain classification and number of plant resistance genes; **(C)** GO classification of all genes; **(D)** KEGG classification of all genes; **(E)** KEGG pathway enrichment analysis; **(F)** GO enrichment analysis.

The PlantTFDB and the PRGdb were used to annotate transcription factors and plant resistance genes, respectively (Sanseverino et al., 2009). A total of more than 40 transcription factors were identified, including several TF families with many members, such as MYB, AP2-EREBP, bHLH, NAC, *etc*. (**Figure 2A**). A total of 13 disease resistance genes were annotated, mainly including the coiled-coil nucleotide-binding leucine-rich repeat (CC-NB-LRR/CNL) domain, NBS domain (N), NBS and LRR (NL) domain, receptor serine-threonine kinase-like and extracellular leucine-rich repeat (RLP) domain, Toll-interleukin receptor-like (TIR) domain, and TIR-NB-LRR/TNL domain (**Figure 2B**). In addition, the GO and KEGG databases were used for gene annotation and functional enrichment analysis. The results of GO and KEGG annotations show that these genes were involved in genetic information processing, cellular processes, metabolic processes, membranes, protein binding, and catalytic activities (**Figures 2C, D**). The GO and KEGG functional enrichment showed that these genes mainly involved in ascorbate and aldarate metabolism, lysine degradation, isoquinoline alkaloid biosynthesis, ubiquitin-mediated proteolysis, glycoside synthesis and degradation, protein processing, and other metabolic pathways and molecular regulation processes (**Figures 2E, F**).

### Gene quantification and time-series analysis

**Figure 3:**
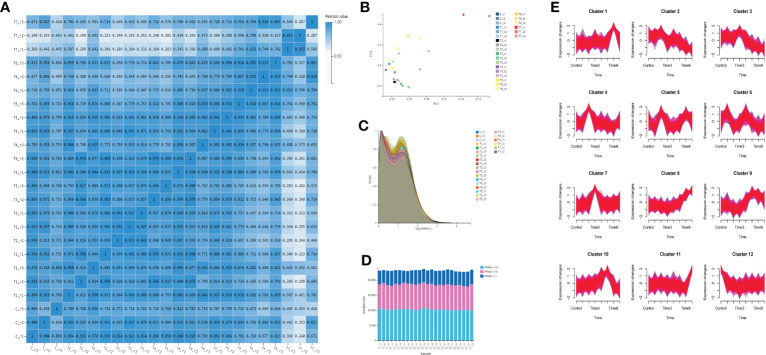
Gene quantification and gene expression analysis. **(A)** correlation analysis among all sample groups; **(B)** principal component analysis among samples; **(C)** gene expression density of all groups; **(D)** gene expression distribution of all samples; and **(E)** time series analysis of all genes.

To confirm the correlation between samples, the Pearson correlation coefficient of the gene amounts were calculated between every two samples (**Table S5**). The gene expression similarity in the three biological replicates of each group is the highest, indicating that the reproducibility of the samples is good. The similarity between the two groups of T5 and T6 is relatively high, and the correlation coefficients are above 0.8%, followed by the two groups of T3 and T4 (**Figure 3A**). The principal component analysis shows that except for the T7 group, the differences among the other groups of samples are not significant (**Figure 3B**). However, some samples between groups, such as T2 and T3 groups, were not completely separated by PCA analysis. The expression density shows that most genes have a similar expression trend, and the expression (log_2_ (FPKM+1)) is mainly distributed between 0 and 2. When the gene abundance is between 0.2 and 1.5, the gene abundance is high (**Figure 3C**). The number of genes with FPKM values more than or equal to 10 is the greatest in each group, followed by those with expression levels between 1 and 10 (**Figure 3D**). Time-series analysis based on a loose clustering algorithm showed that these genes clustered into 12 expression patterns in eight different time periods (**Figure 3E**). The gene expression peak of cluster 1 is at T6, the gene expression peak of cluster 7 is at T3, the gene expression peak of cluster 4 is at T2, and the gene expression peak of cluster 3 is at T3. The gene expression of cluster 8 gradually increased with the MeJA treatment, and the general trend of the gene expression gradually decreased in cluster 8.

### Hierarchical clustering, and functional enrichment analysis of DEGs

**Figure 4:**
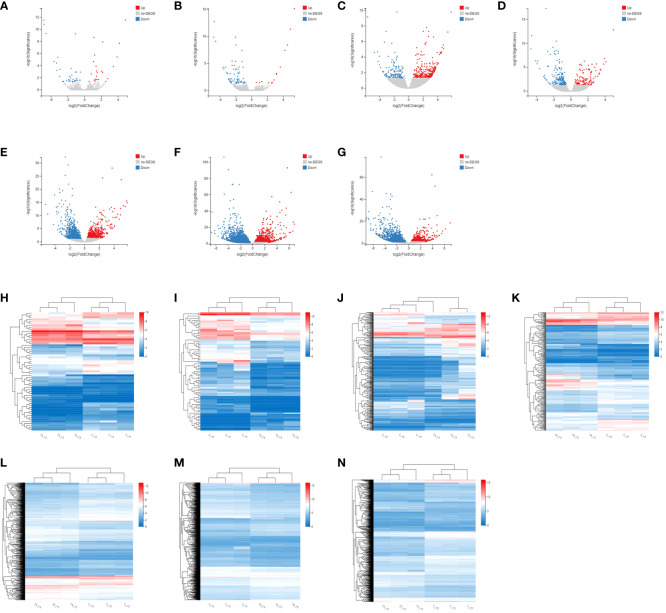
Screening and cluster analysis of DEGs. **(A)** DEGs between Control-vs-Time1; **(B)** DEGs between Control-vs-Time2; **(C)** DEGs between Control-vs-Time3; **(D)** DEGs between Control-vs-Time4; **(E)** DEGs between Control-vs-Time5; **(F)** DEGs between Control-vs-Time6; **(G)** DEGs between Control-vs-Time7; Hierarchical clustering of DEGs in **(H)** Control-vs-Time1; **(I)** Control-vs-Time2; **(J)** Control-vs-Time3; **(K)** Control-vs-Time4; **(L)** Control-vs-Time5; **(M)** Control-vs-Time6; **(N)** Control-vs-Time7.

**Figure 5:**
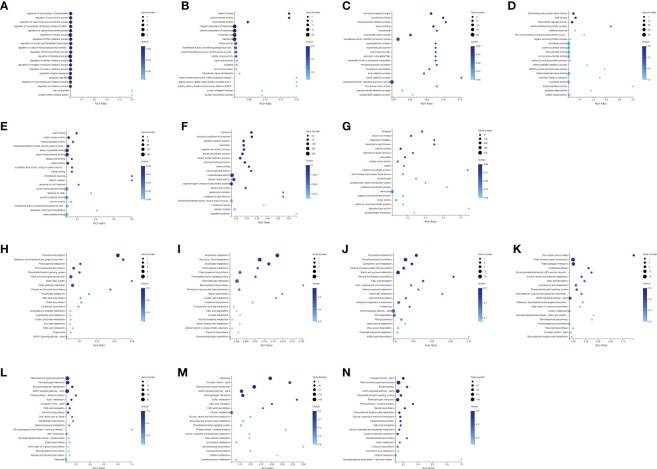
Annotation and functional enrichment of DEGs. GO enrichment analysis of DEGs of **(A)** Control-vs-Time1; **(B)** Control-vs-Time2; **(C)** Control-vs-Time3; **(D)** Control-vs-Time4; **(E)** Control-vs-Time5; **(F)** Control-vs-Time6; **(G)** Control-vs-Time7; KEGG pathway enrichment analysis of DEGs of **(H)** Control-vs-Time1; **(I)** Control-vs-Time2; **(J)** Control-vs-Time3; **(K)** Control-vs-Time4; **(L)** Control-vs-Time5; **(M)** Control-vs-Time6; **(N)** Control-vs-Time7.

Firstly, the DEGs between the CK and the MeJA treatment group were analyzed. The number of DEGs in the Control-vs-Time6 group was at most 4151, including 1730 up-regulated genes and 2421 down-regulated genes. The next group is the Control-vs-Time7 group, with a total of 2427 DEGs. There were 1,504 differential genes in the Control-vs-Time5 group (**Figure S1**). The number of DEGs among the four groups from Control-vs-Time1 to Control-vs-Time4 was lower (**Figures 4A–G**). Heatmap clustering analysis showed that the DEGs between the CK and the MeJA group at different time points showed various patterns. The expression of most genes showed an up-regulation trend under MeJA treatment (**Figures 4H–N**). Control-vs-Time1 and Control-vs-Time2 two groups contained relatively few DEGs; only 16 and 18 DEGs were highly expressed (FPKM > 8). However, most differential genes had moderate expression levels in the Control-vs-Time6 and Control-vs-Time7 groups. Furthermore, GO and KEGG pathway classification and functional enrichment were performed on these DEGs. GO classification showed that molecular functions involving molecular binding and catalytic activity contain the largest number of genes, followed by cellular components involving membrane structure and organelle composition and biological processes such as metabolic processes (**Figures S2A–G**). KEGG pathway classification mainly involves global maps and carbohydrate generation and involves protein folding, sorting, and degradation, as well as genetic information processing such as transcription and translation (**Figures S2H–N**). The GO functional enrichment results indicated that the enriched pathways and biological processes were quite different under different time treatments (**Figures 5A–G**). For example, the Control-vs-Time2 group mainly involves pattern binding, polysaccharide binding, and carbohydrate binding. The Control-vs-Time4 group mainly involves acylphosphatase activity and tetrahydrofolate regulator activity. The Control-vs-Time5 group mainly involves microtubule severing and the katanin complex. The Control-vs-Time6 mainly involves radiation, light stimulus responses and organelle assembly. The Control-vs-Time7 mainly involves cysteine biosynthesis and phosphorylase activity. KEGG pathway functional enrichment indicated that the enrichment of glycosphingolipid biosynthesis in the Control-vs-Time5 and Control-vs-Time7 groups was the most obvious (rich factor ratio close to 1). Followed by the Control-vs-Time6 group, the main pathways involved are monoterpene biosynthesis, sulfur metabolism, circadian rhythm, *etc*. Exceptionally, some secondary metabolic pathways involved in flavone and flavonol biosynthesis, vitamin B6 metabolism, brassinosteroid biosynthesis, glutathione metabolism, *etc*. were also significantly enriched in other groups (**Figures 5H–N**).

### Acquisition of gene sequences and identification of the *bHLHs*

Through NCBI, EnsemblPlants, and TAIR databases, the genome sequences and annotation files of *D. huoshanense, A. thaliana, O. sativa, D. nobile*, and *D. chrysotoxum* were obtained. The established hidden Markov model was used to compare and predict the DhbHLH genes, and the identified bHLH genes were further retrieved and verified using the Pfam, Interpro, and Smart databases. A total of 83 bHLH genes were identified. Further, the physical characteristics of these bHLH candidates were analyzed (**Table S6**). The amino acid length of these bHLHs ranged from 85 to 1689, the relative molecular mass ranged from 9855 to 183707, and the protein isoelectric points ranged from 4.4 to 10.2. The grand average of hydropathicity (GRAVY) ranges from -0.798 to -0.076. The aliphatic index ranges from 57.3 to 103.1.

### Collinearity analysis of the *bHLH* genes

**Figure 6:**
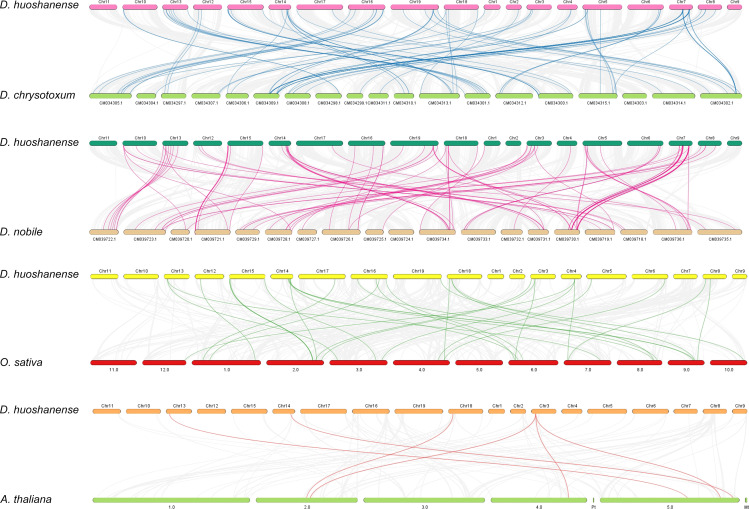
Collinearity analysis of DhbHLH and bHLHs from other species.

To reveal the evolutionary relationship of *bHLH* genes among different species, all the synteny blocks of *D. huoshanense, A. thaliana, O. sativa, D. nobile*, and *D. chrysotoxum* were obtained by collinear analysis (**Figure 6**). The number of gene pairs of *D. huoshanense* and *D. nobile* was the highest, with a total of 92, followed by *D. chrysotoxum*, with 89 gene pairs. *D. huoshanense* had fewer syntenic blocks with *O. sativa* and *A. thaliana*, 31 and 6 blocks, respectively (**Table S7**). The above results are consistent with the genetic relationship between species. It is worth noting that there is synteny between some *DhbHLH* genes and multiple *bHLH* genes of *D. nobile*, and *D. chrysotoxum*. For example, *Dhu000000317* is collinear with *Maker76141, Maker76022, Maker79668*, and *Maker60892* of *D. chrysotoxum*. *Dhu000027551* is collinear with *Maker76141, Maker66639, Maker60892*, and *Maker86412* of *D. chrysotoxum*. *Dhu000009253* is collinear with *Dnobile06G01687.1, Dnobile13G01347.1, Dnobile13G01310.1*, and *Dnobile13G00716.1* of *D. nobile*. *Dhu000022245* is collinear with *Os03t0205300-00* and *Os07t0588400-00*. *Dhu000013850* has collinearity with *AT2G24260.1*, *AT4G30980.2*, and *AT5G58010.1*.

### Phylogenetic analysis of the DhbHLHs and AtbHLHs

**Figure 7:**
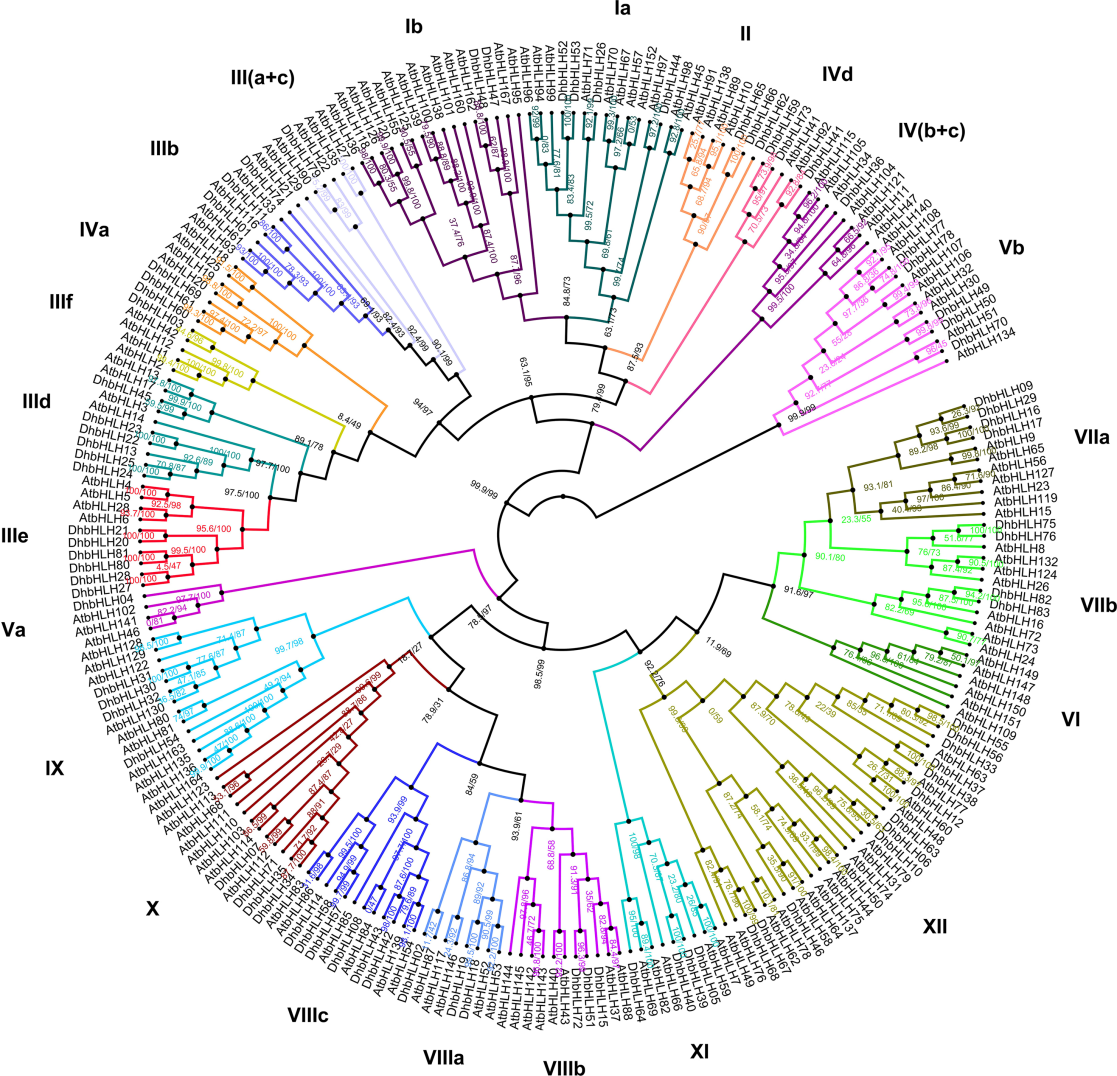
Phylogenetic analysis of the *DhbHLH* and *AtbHLH* genes.

Based on the ML method, a phylogenetic tree for DhbHLHs and AtbHLHs was constructed. According to the *Arabidopsis* nomenclature and the known bHLH classification, the DhbHLH protein was divided into 12 subgroups (groups I–XII) (**Figure 7**). However, Group III can be further divided into six subclades: a, b, c, d, e, and f. Group IV can be divided into four subclades: a, b, c, and d. Group V can be divided into a and b subclades. Group VII can be divided into a and b subclades. Group VIII can be divided into three subclades: a, b, and c. From the ultrafast bootstrap (UFBoot) support and SH-aLRT test results, several DhWRKY proteins have high homology with AtWRKYs. For example, the UFBoot support of DhbHLH26 and AtbHLH71 was 99%, and the SH-aLRT test was 92.1%. The UFBoot support of DhbHLH44 and AtbHLH97 was 100%, and the SH-aLRT test was 97.2%.

### Chromosomal location, conserved motif, and gene structure analysis of the *DhbHLH* gene

**Figure 8:**
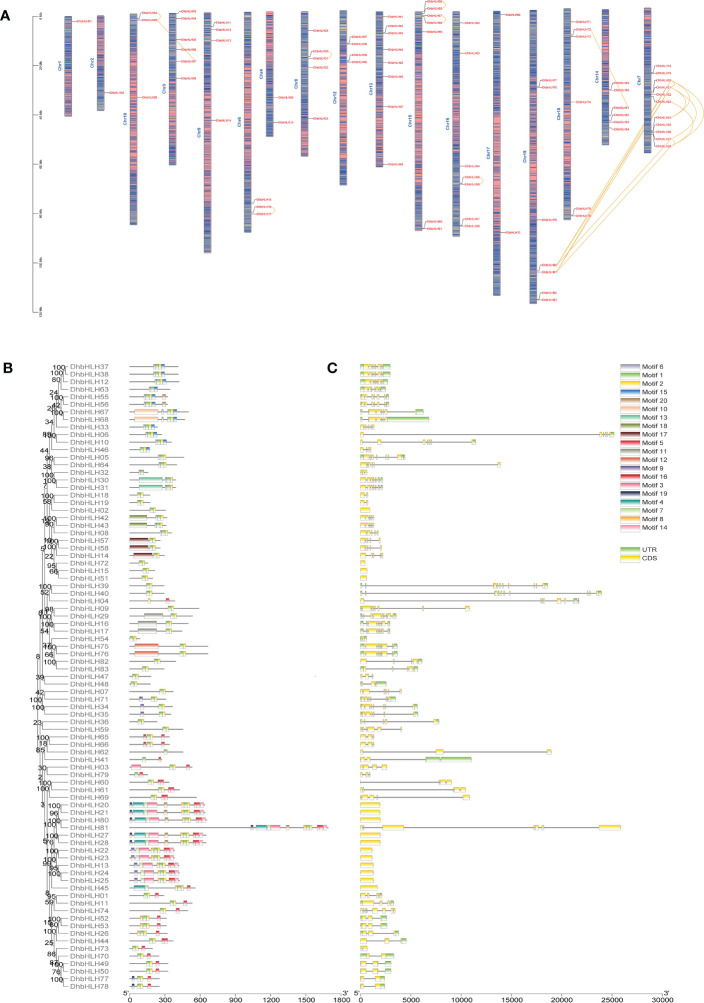
Chromosomal location, conserved motifs, and gene structure of the *DhbHLH* gene. **(A)** Chromosomal distribution of *DhbHLH*. **(B)** Distribution of the conserved motifs of *DhbHLH*. **(C)** The UTRs and exon-intron structures of *DhbHLH*. The chromosome number is represented at the top of each chromosome, and the left scale is in megabases (Mb). There is tandem duplication or segmental duplication between genes.

In order to determine the location of *bHLH* genes on the chromosome of *D. huoshanense*, the structural composition and motif composition of these genes were analyzed. The results showed that *DhbHLH* was unevenly distributed on 17 pseudochromosomes (**Figure 8A**). Chromosome 7 contains the most *bHLH* genes, with 11, followed by chromosome 13 and chromosome 15, with 8 and 7 *bHLH* genes, respectively. In addition, large-scale gene duplication and segmental duplication events of *bHLH* genes existed on different chromosomes. For example, *DhbHLH80* and *DhbHLH81* are associated with *DhbHLH20/21* and *DhbHLH27/28*, respectively. For gene duplications on the same chromosome with a physical distance of less than 100 kb, these *DhbHLH* genes may be obtained through tandem duplication events, like *DhbHLH34* and *DhbHLH35*, *DhbHLH30* and *DhbHLH31*, *DhbHLH57* and *DhbHLH58*, *etc*. Conserved motif analysis showed that the *bHLH* members clustered in the same subgroup had similar motif compositions, and these motifs were regularly distributed among the *bHLH* genes of different subgroups according to the phylogenetic relationship (**Figure 8B**). A total of 20 motifs were identified, among which motifs 1 and 2 are conserved motifs shared by all genes, presumably the conserved domain of bHLH. DhbHLH67/68 from Group XII contains a unique motif 10, and DhbHLH30/31 from Group IX contains a unique motif 13. DhbHLH75/76 from Group VIIb has a unique motif 12. In group VIIIc, DhbHLH30/31 has a unique motif 18, while DhbHLH14/57/58 has a representative motif 17. In Group IIIe, DhbHLH20/21, DhbHLH27/28, and DhbHLH80/81 have a unique motif4, presumably the bHLH-MYC_N domain, but DhbHLH22/23/24/25 from Group IIId do not have this domain. Gene structure analysis showed that all *bHLH* proteins contained exons and introns, and more than half of the genes also contained a 3’ or 5’ UTR (**Figure 8C**). Most genes have multiple exons, but some genes contain only one exon.

### C*is*-acting elements analysis of the *DhbHLH* gene

**Figure 9:**
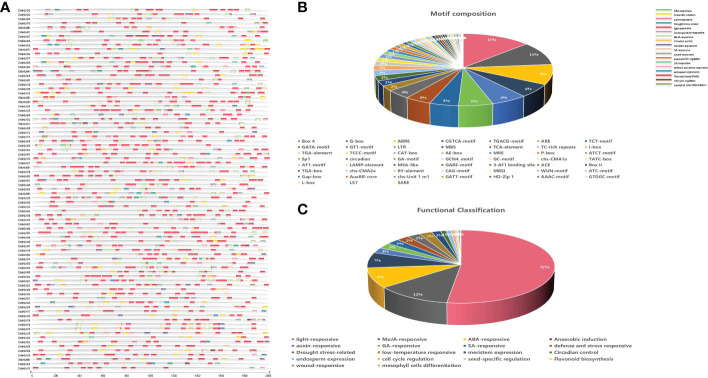
C*is*-acting elements of the *DhbHLH* gene. **(A)** Distribution of all *cis*-acting elements in the upstream 2000 kb of the promoter; **(B)** motif composition and number; **(C)** functional classification and number.

To determine whether *bHLH* genes could be triggered by environmental stress, the regulatory elements within their promoters were examined. The results indicated that the promoters of the majority of genes had a dozen or more cis-acting elements (**Figure 9A**). Some tandemly replicated genes contain *cis*-acting elements of similar composition. Both *DhbHLH34* and *DhbHLH35* have ARE, AE-box, BOX-4, CGTCA-motif, *etc*. Both *DhbHLH30* and *DhbHLH31* contain ABRE, BOX-4, CGTCA-motif, GATA-motif, G-box, I-box, LTR, *etc*. Both *DhbHLH57* and *DhbHLH58* contain ARE, BOX-4, GATA-motif, G-box, GCN4-motif, MBS, P-box, TCT-motif, *etc*. Both *DhbHLH65* and *DhbHLH66* contain ABRE, ARE, BOX-4, G-box, GT1-motif, LTR, RY-element, TCT-motif, *etc*. Both *DhbHLH49* and *DhbHLH50* contain ABRE, ARE, CGTCA-motif, G-box, GT1-motif, LAMP-element, TCCC-motif, *etc*. A total of 52 *cis*-acting elements involved in the growth and stress responses were identified (**Figure 9B**). Box-4 is the most light-responsive component, accounting for 13% of all motifs. This is followed by the G-box motif, which is also associated with light stress. The third most common motif is ABRE, which is mainly related to the ABA-responsive gene. In addition to elements related to stress, some specific *cis*-acting elements were also identified. For example, the CAAAGATATC-motif is a element related to circadian rhythm control, and these elements in the promoter regions of 19 *bHLH* genes were detected. MBSI elements in 6 *bHLH* genes were closely related to the biosynthesis of flavonoids. Functional classification also shows that the number of elements involved in light responsiveness is the largest, followed by MeJA responsiveness, ABA responsiveness, auxin-responsiveness, and other hormone-related regulatory elements (**Figure 9C**).

### Expression profile and correlation analysis of the *DhbHLH* gene under different MeJA treatments

**Figure 10:**
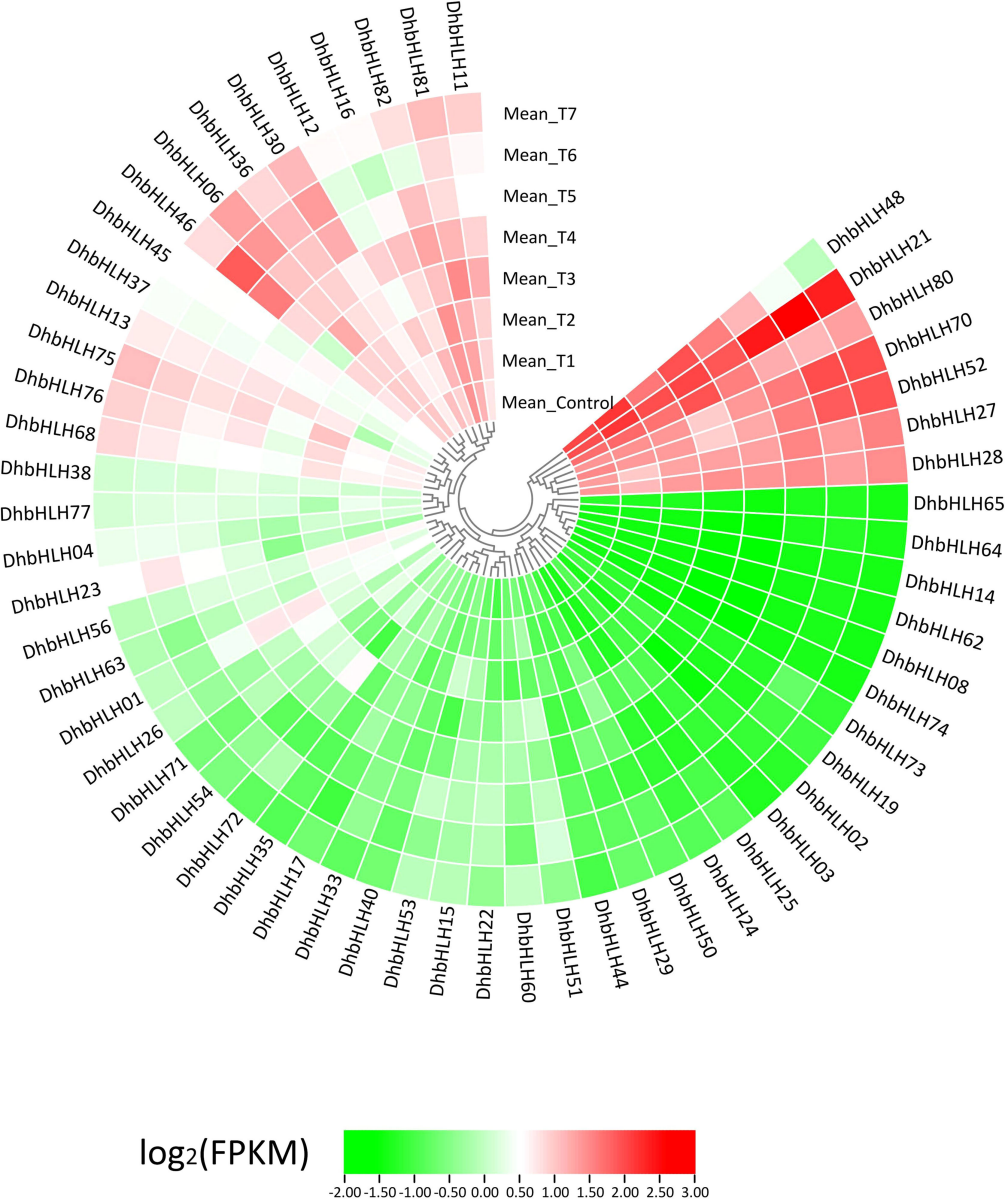
Expression profile of the *DhbHLH* genes under MeJA treatment. All FPKM values are the average of three replicates per group.

**Figure 11:**
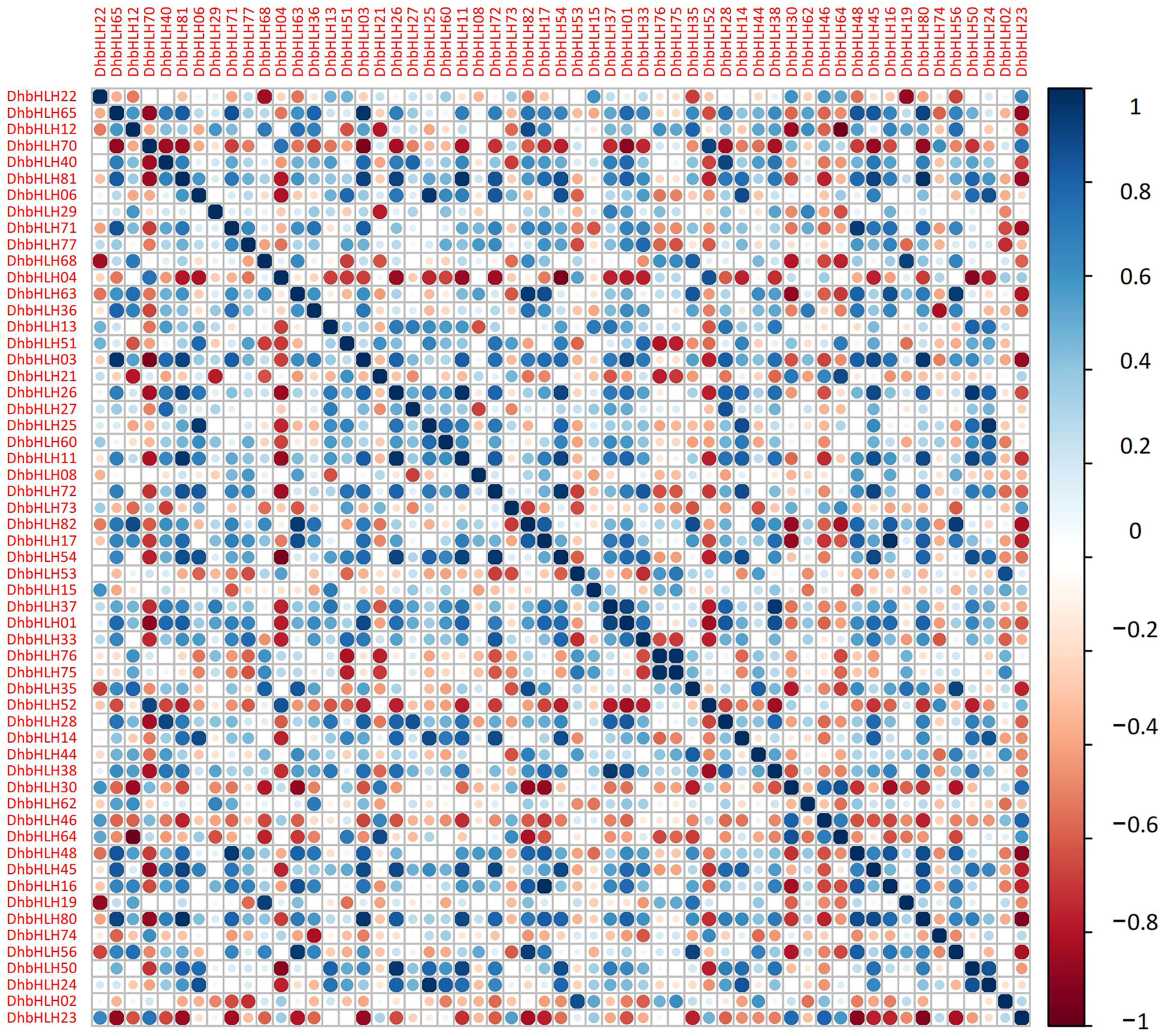
Correlation analysis of the expression levels of main *DhbHLHs*. Blue represents a positive correlation, and red represents a negative correlation.

Using transcriptome sequencing, a batch of DEGs were found in the early stages. To clarify which *DhbHLH* genes were induced or inhibited by MeJA, the heat map clustering analysis at different time points were performed (**Figure 10**; **Table S8**). More than half of the *bHLH* genes were low-expression genes (FPKM<1) throughout the treatment. But there were also some *bHLH* genes, such as *DhbHLH21, DhbHLH27, DhbHLH28, DhbHLH52, DhbHLH70*, and *DhbHL80*, which showed continuous expression throughout the time period. There are also individual genes, such as *DhbHLH46* and *DhbHLH48*, where the expression level of MeJA treatment first increased and then decreased. In addition, a correlation analysis on *bHLH* gene expression levels was performed to ensure which genes might be co-expressed. The blue modules in the figure represent positive correlations, while the red modules represent negative correlations (**Figure 11**). *DhbHLH16* and *DhbHLH17* have the highest correlation (*R^2^ = *0.98), followed by *DhbHLH16* and *DhbHLH17* (*R^2^ = *0.97). The correlation coefficient between *DhbHLH03* and *DhbHLH65* is 0.96. These significantly positively correlated genes may have the characteristics of co-expression. In addition to positively correlated expression patterns, there were also significant negatively correlated expression patterns of some *bHLH* genes, such as *DhbHLH23* and *DhbHLH80* (*R^2^* = -0.93), *DhbHLH23* and *DhbHLH48* (*R^2^* = -0.91).

### Conserved domain, phylogenetic tree, and *q*RT-PCR analysis of the *bHLH* subgroups IIId and IIIe

**Figure 12:**
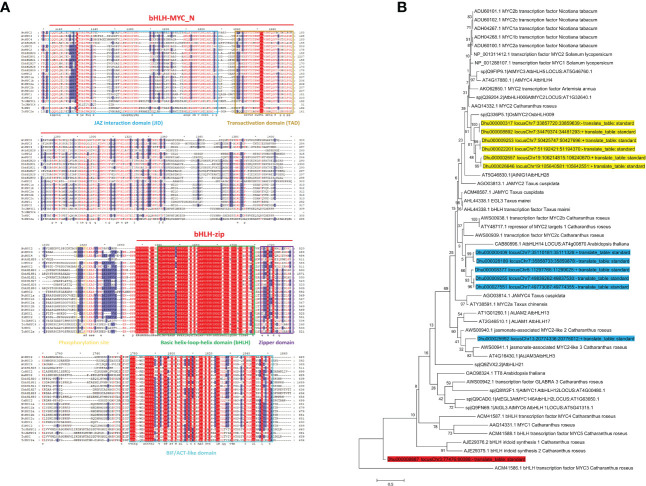
Amino acid sequence alignment and the phylogenetic tree of MYC2-related genes. **(A)** Multiple sequence alignment of DhMYC2 and MYC2-like genes of other species; **(B)** Phylogenetic tree of DhMYC2 and MYC2-like genes of other species. The bootstrap values are indicated in the nodes of the branches. Yellow indicates the MYC2 subclade, blue indicates the JAM subclade, and red is the outgroup. *represents intervals of every 20 amino acidresidues.

Multiple sequence alignment shows that the N-terminus of bHLH subgroups IIId/e has both the bHLH zipper conserved domain and a typical bHLH-MYC domain (**Figure 12A**). The bHLH-MYC_N domain contains JID and TAD, which are responsible for the binding of JAZ to MYC2 and the transcriptional activation of MYC-targeted genes, respectively. DhMYC2 contains GDG motifs, suggesting their ability to bind JAZ proteins (Song et al., 2022b). The presence of RA[K/L]QAQ motifs in DhMYC2 suggests that it is a canonical MYC2 gene. Interestingly, the 469th aa of DhbHLH20 and the 350th aa of AtbHLH28 are both M residues, while this specific aa in other species is a Q residue here. The SDXH motif is the site responsible for the phosphorylation modification of MYC2. The ACT-like domain is widely distributed in the bHLH subgroups IIId/e and forms the βαββαβ secondary structures. The ACT-like domain of DhMYC2 was not highly homologous to the MYC2 genes of several species. A phylogenetic tree was constructed with identified MYC or MYC-like proteins from seven other species to determine which bHLH subgroups IIId/e have potential transcriptional regulatory functions (Stitz et al., 2011). The results indicated that bHLH subgroups IIId/e could be clustered into two major groups (**Figure 12B**). DhMYC2 subfamily members have higher homology with OsMYC2. DhbHLH subgroup IIId (DhbHLH13, DhbHLH23, DhbHLH24, and DhbHLH25) and AtbHLH14 clustered into one group, while DhbHLH subgroup IIId (DhbHLH45) clustered together with AtbHLH3. DhbHLH subgroup IIIf (DhbHLH03) is in a separate branch as an outgroup.

**Figure 13:**
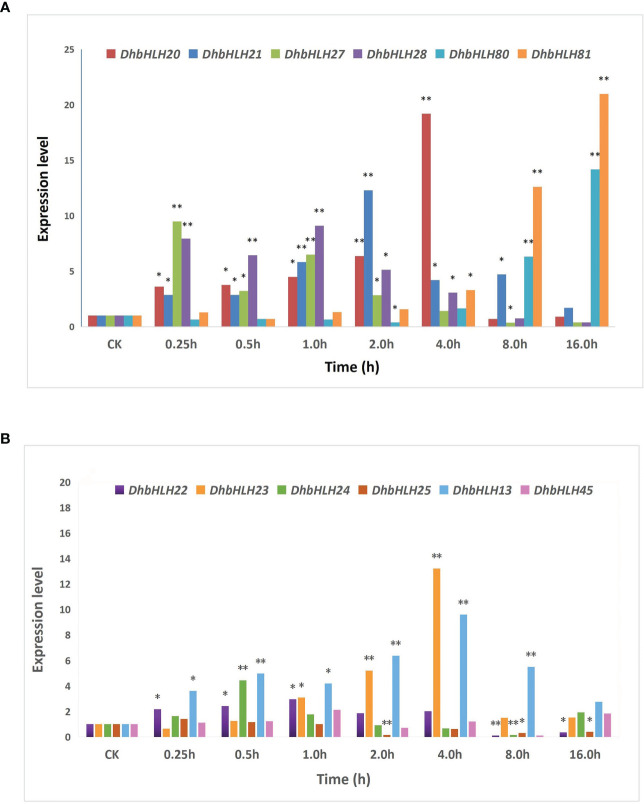
Expression analysis of *bHLH* subgroups IIId and IIIe. **(A)** the expression level of IIIe *DhbHLH* genes; **(B)** the expression level of IIId *DhbHLH* genes. ‘*’P<0.01, ‘**’P<0.001.

To figure out whether *DhbHLH* expression was induced or inhibited by MeJA, the expression pattern of *bHLH* subgroups IIId/e was investigated (**Table S9**). The results indicated that the six members of the *DhMYC2* subfamily showed differential spatiotemporal expression characteristics at different time periods (**Figure 13A**). The expression of *DhbHLH81* and *DhbHLH20* were the highest at 16 and 4 hours of treatment, which were more than 20 times those of the control group. Except for *DhbHLH81*, the other five *DhbHLHs* had the largest peak value during the treatment, and then their expression levels gradually decreased. Compared with the *DhMYC2* subfamily, the expression of *DhbHLH* subgroup IIId genes were generally weaker (**Figure 13B**). The expression of *DhbHLH23* and *DhbHLH13* peaked at the 4th hour and then gradually decreased. Interestingly, the expression of *DhbHLH22, DhbHLH24, DhbHLH25*, and *DhbHLH45* were lower than those of the control group at the 8th hour after treatment. In particular, the expression of *DhbHLH25* and *DhbHLH45* were strongly inhibited by MeJA.

## Discussion

### Comparative transcriptomics of MeJA treatment based on NGS

Comparative transcriptomics technology provides a powerful tool for the discovery of differential genes related to transcriptional regulation (Shoji, 2019). In this study, the time-series transcriptome analysis combined with genome data were integrated to construct the gene regulatory network of JA signaling in *D. huoshanense*. A spatiotemporal expression profile of genes was established under different MeJA treatments. 8,800 new genes were predicted from more than 27,000 identified genes. Nearly 60% of these genes had SE alternative splicing, and A3SS events were less than 20%. Different forms of RNA splicing provide kinetic energy for exon recombination at the transcriptional level (Chini et al., 2016). Through gene annotation and functional enrichment, these DEGs were mainly involved in transcription, translation, protein folding, and the biosynthesis of secondary metabolites (Wang et al., 2021). The JA-mediated gene regulatory network coordinates the spatiotemporal specific activation and co-expression of the genes involved in the aforementioned biological processes (Afrin et al., 2015; Yan et al., 2016). In the initiation stage (within 1 hour), JA preferentially regulated the expression of the JA-biosynthetic genes, primary metabolism-related genes, and some transcription factors. In the effect stage (1-2 hours), enzymes involved in the metabolism of amino acids, fatty acids, sugars, and other substances, as well as genes involved in the plant immune response, began to be expressed in large quantities. During the time-lapse stage (after 2 hours), genes involved in substance transport, hormone transport, and secondary metabolite biosynthesis started gradually activating (Hickman et al., 2017; Zander et al., 2020).

### Identification of *DhbHLH* genes and gene duplication events

bHLH TFs have similar conserved domains of bHLH-MYC N and bHLH-zip, allowing orthologous genes of many species to preserve a high degree of homology. In this study, DhbHLHs maintained high homology with many members of AtbHLH (SH-aLRT ≥ 80% and UFBoot ≥ 95%), especially branches on IIIb and Ia subclades, such as AtbHLH116 and DhbHLH11, AtbHLH71 and DhbHLH26, AtbHLH97 and DhbHLH44, *etc*. (Guindon et al., 2010; Minh et al., 2013). The CDS sequence of the *DhbHLH* gene contains multiple exon and intron structures, and some genes also contain a 3’ or 5’ UTR. The UTR structure is generally considered to be related to gene transcription, but its sequence cannot be translated into protein (Thiel et al., 2021). *DhbHLH62* and *DhbHLH68* have a particularly long 3’ UTR, and their role in gene transcription and expression needs further study. Several studies have shown that two WGDs occurred in the orchid lineage (Zhang et al., 2017; Song et al., 2022b). *Apostasia*, *Dendrobium*, and *Phalaenopsis* all experienced a WGD recently, which may have arisen around the *K/Pg* boundary. Putative peaks in the early *Ks* age distribution could mean that monocot ancestors went through more WGD events in the past (Cai et al., 2015; Zhang et al., 2017). Here, 17 chromosomes contained imbalanced duplicates of these genes. Collinearity analysis showed that some *bHLH* genes underwent WGD or segmental duplication. The *bHLH* genes with similar distances (< 100kb) on the same chromosome were doubled and expanded by paralogous genes through tandem duplication. It acts as a catalyst for the development of new genes.

### *Cis*-acting elements and expression profile analysis of *DhbHLHs*

*Cis*-acting elements are motifs on the promoter region that are triggered by the external environment and typically have tissue- and time-specific transcriptional regulation (Zander et al., 2020). Here, a series of elements involved in hormone signal transduction, cell cycle regulation, circadian rhythm, abiotic stress, and secondary metabolism were identified. More than half of the elements associated with photoperiod control and ABA responsiveness. These elements include the Box 4 motif, the G-box motif, the AREB element, the CGTCA motif, the ARE element, *etc*. Using comparative transcriptomics, a collection of differentially expressed genes was identified to determine which *WRKY* genes can be activated by MeJA. A series of key genes involved in the JA signaling pathway were identified. Only a few *bHLH* genes, such as *DhbHLH21, DhbHLH70, DhbHLH52*, and *DhbHLH27*, were up-regulated in response to MeJA treatment, as shown by heatmap clustering and expression profiling. Previous studies showed that CrORCA3 regulates the JA-responsive genes expression in the biosynthesis of terpenoid indole alkaloids (Zhang et al., 2011). CrMYC2 has the capacity to bind to the promoter of *ORCA3*, hence modulating its mRNA expression (Paul et al., 2017). The MeJA-induced gene from *Solanum lycopersicum* (*SlJIG*) was dramatically induced by MeJA treatment (Cao et al., 2020). *LjbHLH7* regulates the production of cyanogenic glucosides by directly activating the expression level of the *CYP79D3* gene (Chen et al., 2022).

### Expression pattern and function of the IIId/e subgroup *bHLH* genes

Studies have shown that bHLH subgroups IIId/e have unique functions in the JA signaling (Zhao et al., 2021). In this study, homologous sequence alignment and phylogenetic analysis were used to screen out *bHLH* proteins that may have transcriptional activation functions. DhbHLH subgroups IIId/e all contain a typical MYC2 conserved domain, namely bHLH-MYC_N (Liu et al., 2022). The phylogenetic tree indicated that the *bHLH* subgroups IIId and IIIe genes were further classified into two branches, namely the *JASMONATE-ASSOCIATED MYC2-LIKE* (*JAM*) subclade and the *MYC2* subclade. In *A. thaliana*, *bHLH* subgroup IIIe include *MYC2*, *MYC3*, *MYC4*, and *MYC5*, which mainly positively regulate the expression of JA-responsive genes and disease resistance responses (Fernández-Calvo et al., 2011; Niu et al., 2011; Qi et al., 2015b; Song et al., 2017). However, *bHLH* subgroup IIId like *bHLH17/JAM1*, *bHLH13/JAM2*, *bHLH3/JAM3*, and *bHLH14* function as repressors to antagonistically regulate JA responses (Nakata et al., 2013; Sasaki-Sekimoto et al., 2013; Song et al., 2013; Fonseca et al., 2014; Qi et al., 2015b). Six DhbHLH subgroup IIId genes with AtbHLH14, AtbHLH13, AtbHLH17, and AtbHLH3 clustered in one branch, suggesting they could play a negative regulatory role in JA signaling. The DhbHLH subgroup IIIe clustered in a branch with OsbHLH009 and CrMYC2, indicating that they may upregulate the expression of JA-responsive genes.

## Conclusions

In this study, A total of 57 *DhbHLH* members were screened out using a large-scale genome identification. Comparative genomics revealed multiple duplication events of the *bHLH* gene in the *D. huoshanense* genome, which partially explained why the *bHLH* gene’s expansion was obtained through WGD and segmental duplication, as evidenced by collinearity analysis. Thousands of DEGs involved in the regulation of JA signaling were screened out using comparative transcriptomics. These *bHLH* genes showed different expression patterns, more than half of which were low-expression genes. Only a few genes were strongly induced by MeJA. Through the analysis of *cis*-acting elements, it was indicated that more than half of the elements are related to light signal, hormone signaling, and other abiotic stresses. There are also a small number of elements related to the cell cycle, circadian rhythm, and secondary metabolism. The *q*RT-PCR results showed that IIId and IIIe *DhbHLH* subgroups had distinct expression patterns. *DhbHLH20*, *DhbHLH32*, and *DhbHLH81* from the *DhbHLH* subgroup IIIe were strongly induced by JA, but the expression of *DhbHLH81* was somewhat delayed (peaking at 16 hours). The expression patterns of the IIIe *DhbHLH* subgroup were relatively consistent. The expression of the core JA-responsive genes, such as *DhbHLH13* and *DhbHLH23*, both peaked at the fourth hour. The research would provide scientific tools for the discovery of the *bHLH* genes in other *Dendrobium* species.

## Data availability statement

The data presented in the study are deposited in the NCBI GenBank and NGDC GSArepository, accession number PRJNA597621 and CRA006607, respectively. 

## Author contributions

CS, YZ and XH discussed the writing plan. CS, XH, WZ and YW drafted the manuscript. CS and IS edited the manuscript. CS, FZ and JD conducted the experiment. CS, CJ, GL, and LL analyzed the data. CS, XH and CC acquired the funding. All authors contributed to the article and approved the submitted version.

## Supplementary material

Supplementary Figure 1 Number of DEGs among all sample groups

Supplementary Figure 2 Classification of DEGs. GO classification of DEGs of (A) Control-vs-Time1; (B) Control-vs-Time2; (C) Control-vs-Time3; (D) Control-vs-Time4 (E) Control-vs-Time5; (F) Control-vs-Time6; (G) Control-vs-Time7; KEGG pathway classification of DEGs of (H) Control-vs-Time1; (I) Control-vs-Time2; (J) Control-vs-Time3; (K) Control-vs-Time4; (L) Control-vs-Time5; (M) Control-vs-Time6; (N) Control-vs-Time7

Supplementary Table 1 Reference genome alignment ratios for each group.

Supplementary Table 2 Reference gene alignment ratios for each group.

Supplementary Table 3 Number of known and novel genes.

Supplementary Table 4 Alternative splicing classes and gene numbers for each group.

Supplementary Table 5 Correlation coefficients among different sample groups.

Supplementary Table 6 The physical characteristics of identified *bHLH* genes from the *D. huoshanense* genome.

Supplementary Table 7 The gene pairs between *DhbHLH* and *bHLHs* of other four species.

Supplementary Table 8 The FPKM values of *DhbHLH* used for expression profiling.

Supplementary Table 9 The primers of the *q*RT-PCR of the *DhbHLH* genes.
