# Supplementary figures and images for: The spatiotemporal profile of Dendrobium huoshanense and functional identification of bHLH genes under exogenous MeJA using comparative transcriptomics and genomics
Source: Front Plant Sci. 2023 May 10;14:1169386. doi: 10.3389/fpls.2023.1169386 (PMC10206334; doi:10.3389/fpls.2023.1169386)

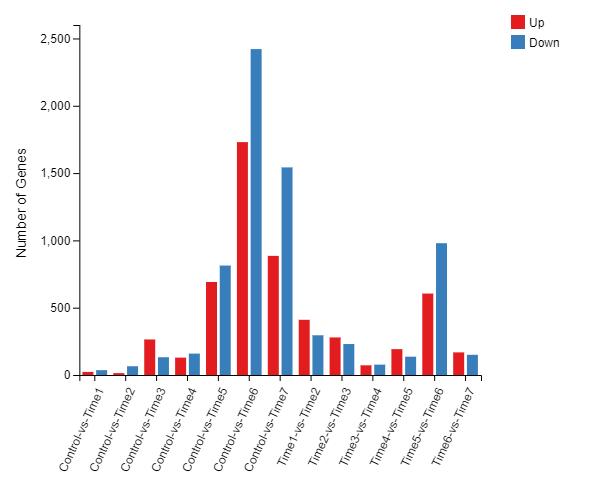

Supplement: Supplementary Figure 1 — Number of DEGs among all sample groups [file Image_1.jpeg]

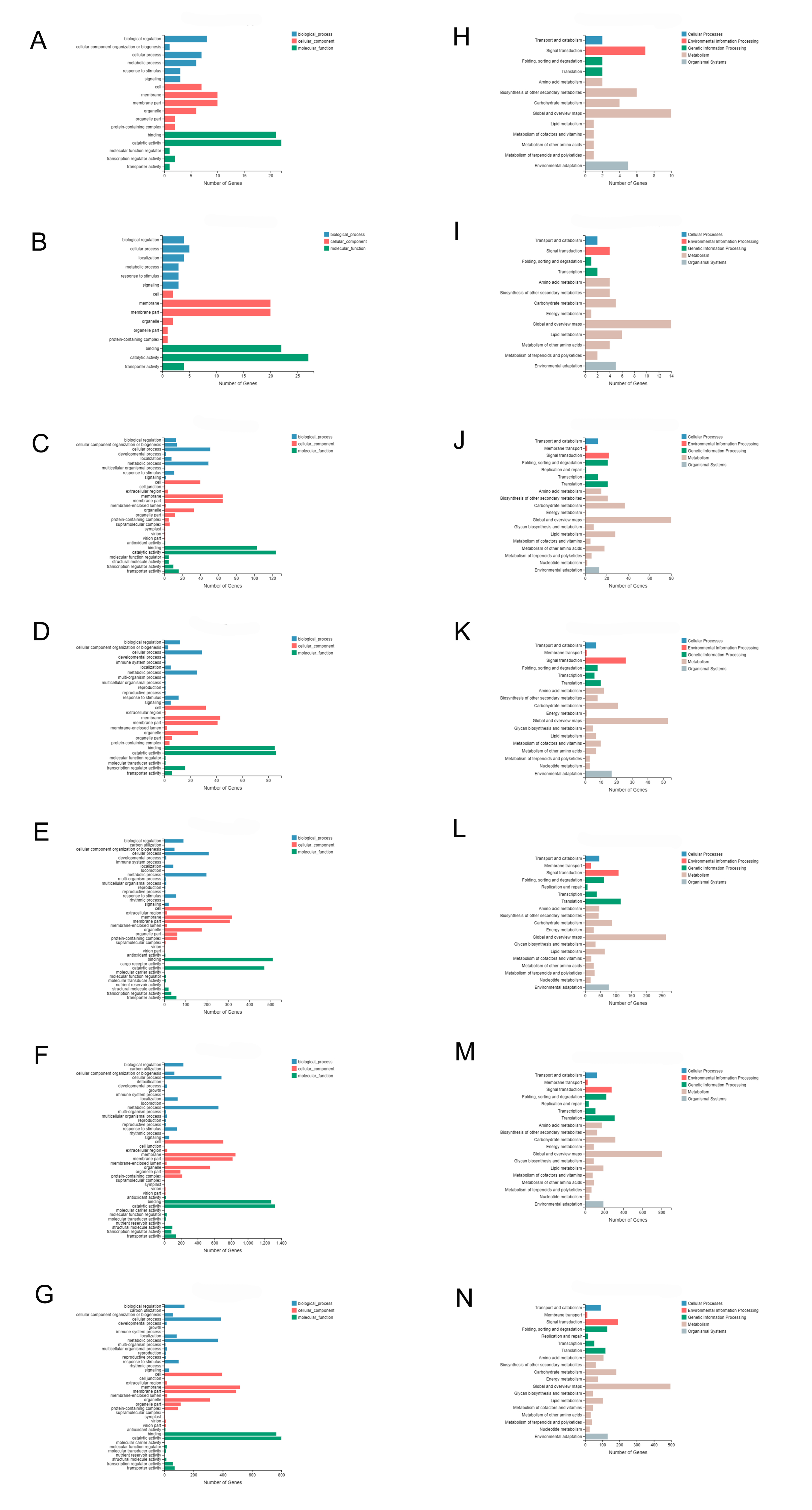

Supplement: Supplementary Figure 2 — Classification of DEGs. GO classification of DEGs of (A) Control-vs-Time1; (B) Control-vs-Time2; (C) Control-vs-Time3; (D) Control-vs-Time4 (E) Control-vs-Time5; (F) Control-vs-Time6; (G) Control-vs-Time7; KEGG pathway classification of DEGs of (H) Control-vs-Time1; (I) Control-vs-Time2; (J) Control-vs-Time3; (K) Control-vs-Time4; (L) Control-vs-Time5; (M) Control-vs-Time6; (N) Control-vs-Time7 [file Image_2.jpeg]
